# Association of Cardiovascular Risk Factors and Metabolic Syndrome with non-alcoholic and alcoholic fatty liver disease: a retrospective analysis

**DOI:** 10.1186/s12902-021-00758-x

**Published:** 2021-05-01

**Authors:** A. L. Han

**Affiliations:** Department of Family Medicine, Wonkwang University Hospital, Sinyong-dong, 344-2 Iksan, Jeollabuk-do Republic of Korea

**Keywords:** Non-alcoholic fatty liver, Alcoholic fatty liver, Cardiovascular risk factor, Metabolic syndrome

## Abstract

**Background:**

Although many studies on non-alcoholic fatty liver disease (NAFLD) are underway worldwide, and several existing studies have investigated the association between NAFLD and cardiovascular risk factors, studies comparing NAFLD and alcoholic fatty liver disease (AFLD) are scarce. This study aimed to evaluate differences between the incidence of cardiovascular risk factors and metabolic syndrome in NAFLD and AFLD.

**Methods:**

A retrospective analysis of 913 patients who underwent abdominal computed tomography (CT) was performed to compare the incidence of cardiovascular risk factors and metabolic syndrome between NAFLD and AFLD. Subjects were divided into three groups based on criteria: healthy (*n* = 572), NAFLD (*n* = 295), and AFLD (*n* = 46). The healthy group had no liver disease. NAFLD was defined as fatty liver diagnosed on CT and drinking less than 140 g/week for men or 70 g/week for women. AFLD was defined as fatty liver diagnosed on CT and drinking more than 140 g/week for men or 70 g/week for women. We compared the incidence of cardiovascular risk factors and metabolic syndrome between the three groups. The relationship between each group and the metabolic syndrome risk was analyzed through multivariate logistic regression analysis.

**Results:**

No significant differences in several cardiovascular risk factors were observed between the NAFLD and AFLD groups. Upon analyzing the metabolic syndrome status in each group after making appropriate adjustments, the odds ratios (ORs) in the NAFLD (OR = 2.397, *P* = 0.002) and AFLD groups (OR = 4.445, *P* = 0.001) were found to be significantly higher than that in the healthy group; the incidence rate of metabolic syndrome was similar in the NAFLD and AFLD groups.

**Conclusions:**

Both the NAFLD and AFLD groups had more cardiovascular risk factors and higher metabolic syndrome risk than the healthy group. Thus, the prevention of fatty liver disease, regardless of the specific type, should involve the identification of cardiovascular and metabolic syndrome risk factors. If abdominal CT reveals a fatty liver, whether NAFLD or AFLD, the risk of cardiovascular disease and metabolic syndrome should be assessed.

**Supplementary Information:**

The online version contains supplementary material available at 10.1186/s12902-021-00758-x.

## Background

Fatty liver disease is defined as the accumulation of fat (mainly triglycerides [TG]) in hepatocytes that accounts for > 5% of the total liver weight. It includes benign steatosis and steatohepatitis, which can cause cirrhosis [1]. Fatty liver disease is largely divided into alcoholic fatty liver disease (AFLD) and non-alcoholic fatty liver disease (NAFLD), depending on the drinking status of the patient. Distinguishing between the two types cannot be done clinically, biochemically, or by liver tissue biopsy, but only by determining the quantity of alcohol intake [1].

Fatty liver disease can be diagnosed using imaging techniques such as ultrasonography, computed tomography (CT), and magnetic resonance imaging (MRI) [2]. Although the most accurate method for diagnosis is liver tissue biopsy, it is clinically difficult to perform. Therefore, assessment of the severity of fatty liver disease in clinical practice is mostly dependent on the clinical experience of the diagnosing physician [3].

Alcoholic fatty liver is caused by excessive drinking. This is due to increased TG synthesis and increased influx of fatty acids from peripheral tissues into the liver during drinking, when the release of lipoproteins into the blood and the oxidation of fatty acids are poor [4]. While the condition can be alleviated by abstinence from alcohol, it can also progress to hepatitis, cirrhosis, and hepatocellular carcinoma [5]. Meanwhile, NAFLD has pathological characteristics similar to those of AFLD but occurs in nondrinkers and can progress to chronic liver diseases [6].

As the number of health screening examinees has been increasing in recent years, along with an increased interest in health, the number of people diagnosed with fatty liver disease has also increased. Patients should be informed of the risks associated with fatty liver disease and the relationship of the disease with cardiovascular diseases, in order to help them understand the lifestyle changes needed to reduce risk factors, and should be provided with information about the treatment of this disease.

Obesity, diabetes, and hyperlipidemia are known factors that increase the risk of AFLD [7], which is known to increase the prevalence rate of metabolic syndrome and disorders [8, 9]. Although many studies on NAFLD are underway worldwide, and several existing studies have investigated the association between NAFLD and cardiovascular risk factors, studies comparing NAFLD and AFLD are scarce. Therefore, in this study, we investigated the morbidity rate of AFLD and NAFLD in male health-screening examinees, while assessing their pathological and behavioral characteristics. We performed a comparative analysis of the association between each cardiovascular risk indicator, and the differences between these indicators, by dividing the subjects into healthy, AFLD, and NAFLD groups.

## Methods

### Study aim

This study investigated the morbidity rate of AFLD and NAFLD in male health-screening examinees, while assessing their pathological and behavioral characteristics.

### Study subjects

Study subjects older than 19 years of age were selected from among male and female city residents who underwent examinations at a university hospital health promotion center between March 2013 and March 2018. Based on the assumption of a significant difference in drinking habits between urban residents and the relatively fewer rural residents (Eop or Myon), the latter were excluded to keep the study conditions relatively homogeneous. The medical records of examinees who underwent CT were retrospectively reviewed in this study. Those who met the following criteria were also excluded from the study: incomplete records, leukocyte count > 10.0 × 10^3^/μL, acute hepatitis-related test findings, hepatitis B surface antigen or hepatitis C virus antibody positive, abnormal thyroid function test findings, abdominal ultrasonography findings suggestive of diffuse or local liver diseases, creatinine level > 1.3 mg/dL, or a history of cancer. In addition, those with hypertension, diabetes, rheumatoid arthritis, asthma, rhinitis, ischemic heart diseases and myocardial infarction, cerebral infarction and hemorrhage, and thyroid diseases, or those receiving hormone replacement therapy were also excluded. Participants with high-sensitivity C-reactive protein (hsCRP) levels > 10 mg/dL were excluded because they were suspected of having active inflammation or tissue damage, such as acute infection or systemic inflammation [10]. In addition, patients with normal abdominal ultrasonographic findings, but with aspartate aminotransferase (AST), alanine aminotransferase (ALT), and gamma-glutamyl transpeptidase (*ϓ*-GT) levels > 100 IU/L were excluded. As a result, 913 participants were included in the final analysis. This study followed the ethical standards laid out in the Declaration of Helsinki. The study was approved by the Clinical Trial Screening Committee of Wonkwang University Hospital (institutional review board approval number: 201609-HR-097), and informed consent was obtained verbally from all participants.

### Study methods

Through self-administered questionnaires and interviews with the attending physicians at the health promotion center, factors such as disease history, ongoing treatments and medications for existing diseases, drinking and smoking habits, and exercise habits were investigated. The types of alcohol and the average amount consumed per week were surveyed. By dividing the participants into current smokers and nonsmokers, the smoking status of the participants was also surveyed. The average weekly exercise frequency was also recorded.

#### Anthropometric measurements and blood pressure

Height and weight were measured using an automatic height scale, and body mass index (BMI) was calculated by dividing weight by the square of height (kg/m^2^). Waist circumference was measured according to the level recommended by the World Health Organization at the middle (halfway) point between the lowest rib and iliac crest on the CT image. Blood pressure was measured using an automatic blood pressure monitor after maintaining stable pressure for > 10 min, and the average of two measurements was recorded.

#### Blood tests

Venous blood was collected at 10:00 AM, after more than 12 h of fasting. Fasting plasma glucose, total cholesterol, high-density lipoprotein cholesterol, TG, low-density lipoprotein cholesterol, AST, ALT, *ϓ*-GT, and hsCRP levels were measured.

#### Abdominal CT

In order to avoid examiner bias, all of the data were reconfirmed by one medical imaging specialist who was blinded to the patients’ characteristics and study aims. CT examinations of the abdomen were performed using the Somatom Definition (Siemens Medical Solutions, Forchheim, Germany). Steatosis was confirmed by either a liver attenuation < 40 Hounsfield Units (HU) or a liver attenuation of at least 10 HU less than that of the spleen [11, 12].

#### Classification of study groups

**Fig. 1:**
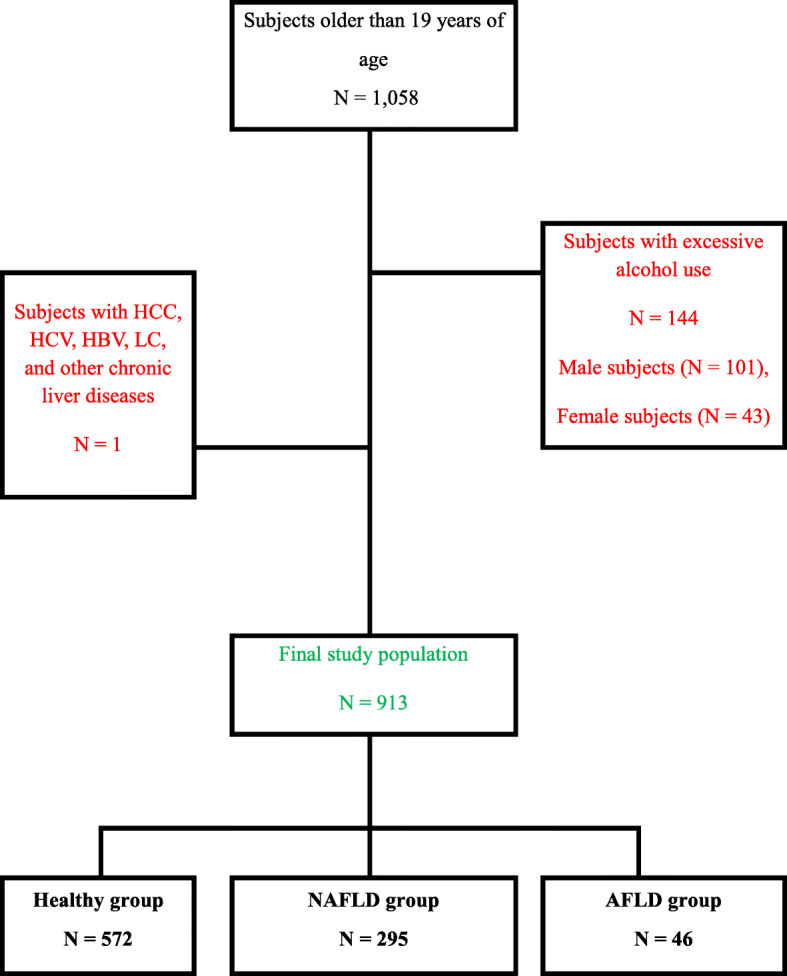
Flow diagram of subjects in this study. HCC, hepatic cellular carcinoma; HCV, hepatitis virus C; HBV, hepatitis virus B; LC, liver cirrhosis; NAFLD, non-alcoholic fatty liver disease; AFLD, alcoholic fatty liver disease

The healthy group comprised examinees who showed normal liver echo on abdominal CT; whose AST, ALT, and *ϓ*-GT values were normal in the liver function tests; and who drank alcohol at a rate of less than 140 g/week for men or 70 g/week for women [13]. The NAFLD group comprised examinees who were diagnosed with fatty liver disease according to abdominal CT results and drank less than 140 g/week for men and 70 g/week for women, regardless of the liver function test values [14, 15]. Meanwhile, the AFLD group comprised participants who drank more than 140 g/week for men or 70 g/week for women and were not obese (Fig. 1).

### Statistical analysis

SPSS for Windows version 26.0 (SPSS Inc., Chicago, IL, USA) was used for statistical analysis. A comparative analysis between non-continuous variables was performed using the chi-square test. The comparative analysis between continuous variables was performed using one-way analysis of variance. The Scheffe test was used for post hoc testing of differences between the groups. The analysis of covariance was used to analyze the average difference between the groups for the following factors: waist circumference, blood pressure, fasting plasma glucose, total cholesterol, high-density lipoprotein cholesterol, TG, low-density lipoprotein cholesterol, and hsCRP levels, after adjusting for age, BMI, daily smoking quantity, and weekly exercise frequency. The differences between the groups were confirmed using a simple and repeated method of contrast testing. The relationship between each group and the metabolic syndrome was analyzed through multivariate logistic regression analysis after adjusting for age, daily smoking quantity, and weekly exercise frequency. Statistical significance was set at *P* < 0.05.

## Results

### General patient characteristics

The total numbers of involved participants were as follows: 572 (62.7%), healthy group; 295 (32.3%), NAFLD group; and 46 (5.0%), AFLD group. The average ages were 52.5 ± 10.4 in the healthy group, 52.7 ± 9.7 in the NAFLD group, and 47.4 ± 9.1 in the AFLD group. The numbers of male subjects were 298 (52.1%) in the healthy group, 210 (71.2%) in the NAFLD group, and 39 (84.8%) in the AFLD group. The average BMI was higher in the AFLD group than in both the NAFLD and healthy groups. Significant differences in AST, ALT, and *ϓ*-GT levels were observed between the groups; the AST and *ϓ*-GT levels in the NAFLD group were higher than those in the healthy group. The ALT level was higher in the NAFLD and AFLD groups than in the healthy group. Both the AFLD and NAFLD groups had a larger waist circumference than the healthy group, whereas no significant difference was found between the AFLD and NAFLD groups. Significant differences were found in the average weekly exercise frequency in the following decreasing order: the AFLD, healthy, and NAFLD groups (Table 1).

**Table 1:** Baseline characteristics of the study subjects

Characteristic	Healthy (Group A)	NAFLD (Group B)	AFLD (Group C)	P	Scheffe test
Total number of subjects	572 (62.7)	295 (32.3)	46 (5.0)	ND	ND
Age (years)	52.5 ± 10.4	52.7 ± 9.7	47.4 ± 9.1	0.003	b < a, c
Sex					
Male	298 (52.1)	210 (71.2)	39 (84.8)		
female	274 (47.9)	85 (28.8)	7 (15.2)	< 0.0001	
BMI (kg/m^2^)	23.8 ± 2.8	25.9 ± 3.5	33.5 ± 47.0	< 0.0001	a, b < c
AST (IU/L)	30.3 ± 21.7	37.0 ± 37.6	36.6 ± 14.6	0.002	a < b
ALT (IU/L)	26.9 ± 23.8	38.9 ± 29.5	39.2 ± 24.9	< 0.0001	a < b, c
*ϓ*-GT (IU/L)	35.1 ± 44.7	68.1 ± 219.5	75.2 ± 57.7	0.001	a < b
Waist circumference (cm)	81.7 ± 9.1	87.8 ± 9.9	89.7 ± 9.5	< 0.0001	a < b, c
Smoking status					
None	436 (76.2)	209 (70.8)	14 (30.4)	< 0.0001	
Ex-smoker	3 (0.5)	1 (0.3)	0 (0.0)	< 0.0001 0.040	b < a < c
Smoker	133 (23.3)	85 (28.8)	32 (69.6)		
Exercise (frequency/week)	1.0 ± 1.8	0.8 ± 1.6	1.5 ± 2.0		
Fatty liver grade					
Mild	ND	144 (48.8)	22 (47.8)	< 0.0001	
Moderate	ND	100 (33.9)	15 (32.6)	< 0.0001	
Severe	ND	51 (17.3)	9 (19.6)		

Data are presented as number (%) or mean ± standard deviation

*Abbreviations*: *NAFLD* non-alcoholic fatty liver disease, *AFLD* alcoholic fatty liver disease, *BMI* body mass index, *AST* aspartate aminotransferase, *ALT* alanine aminotransferase, *ϓ*-*GT* gamma-glutamyl transferase, *ND* not done, *NA* not available

^a^Continuous variables were analyzed using one-way analysis of variance, and the post hoc test was performed using the Scheffe test. Categorical variables were analyzed using the chi-square test

### Comparison of cardiovascular risk indicators

Significant differences were found between the groups in terms of average systolic blood pressure, diastolic blood pressure, fasting plasma glucose level, total cholesterol level, TG level, high-density lipoprotein level, low-density lipoprotein level, and hsCRP level. In the comparative analysis between the groups through post hoc tests, no significant difference was found between the NAFLD and AFLD groups in terms of systolic blood pressure, diastolic blood pressure, fasting plasma glucose level, total cholesterol level, high-density lipoprotein level, TG level, low-density lipoprotein level, and hsCRP, but the values from either group were higher than those of the healthy group.

Systolic blood pressure was higher in the NAFLD group than in the healthy group, and the diastolic blood pressure was higher in the NAFLD and AFLD groups than in the healthy group. The fasting plasma glucose level was highest in the AFLD group, followed by the NAFLD group, and lowest in the healthy group. High-density cholesterol was lower in the NAFLD group than in the healthy group. TG levels were higher in the NAFLD and AFLD groups than in the healthy group. Low-density cholesterol was higher in the NAFLD group than in the healthy group. The hsCRP level was higher in the AFLD group than in the healthy and NAFLD groups (Table 2).

**Table 2:** Comparison between the cardiovascular risk factors according to the type of fatty liver

Cardiovascular Risk Factor	Healthy (Group A)	NAFLD (Group B)	AFLD (Group C)	P^a^	Scheffe test	P^b^ A vs. B	P^c^ A vs. C	P^d^ B vs. C
SBP (mm Hg)	121.3 ± 12.6	125.4 ± 11.8	124.5 ± 13.1	< 0.0001	a < b	< 0.0001	0.242	0.909
DBP (mm Hg)	74.8 ± 9.5	78.4 ± 10.2	79.7 ± 9.5	< 0.0001	a < b, c	< 0.0001	0.005	0.691
Glucose (mg/dL)	93 ± 23.3	97.5 ± 25.9	103.4 ± 25.9	0.002	a < b < c	0.033	0.020	0.314
TC (mg/dL)	199.4 ± 35.2	206.1 ± 43.7	207 ± 41.9	0.037	–	0.056	0.434	0.987
HDL-C (mg/dL)	56.9 ± 13.1	53.9 ± 14.4	53.8 ± 14.2	0.005	b < a	0.008	0.327	0.999
TG (mg/dL)	101 ± 64.8	138.7 ± 140.9	147.4 ± 81.6	< 0.0001	a < b, c	< 0.0001	0.008	0.852
LDL-C (mg/dL)	118.8 ± 32.3	125.5 ± 38.1	128.5 ± 38.3	0.009	a < b	0.024	0.187	0.868
hsCRP (mg/dL)	1.6 ± 3.5	1.9 ± 3.6	3.3 ± 6.3	0.018	a, b < c	0.576	0.021	0.087

*Abbreviations*: *NAFLD* non-alcoholic fatty liver disease, *AFLD* alcoholic fatty liver disease, *SBP* systolic blood pressure, *TC* total cholesterol, *TG* triglyceride, *DBP* diastolic blood pressure, *HDL-C* high-density lipoprotein cholesterol, *LDL-C* low-density lipoprotein cholesterol, *hsCRP* high-sensitivity C-reactive protein, *NA* not available. Data are presented as mean **±** standard deviation

^a^The *P* values were calculated using one-way analysis of variance, and the post hoc test was performed using the Scheffe test

^b^The *P* values were calculated using the analysis of covariance after adjusting for age, body mass index, smoking quantity per day, and weekly exercise frequency per week

^c^The *P* values were calculated using the simple contrast test

^d^The *P* values were calculated using the repeated contrast test

### Association with metabolic syndrome

In the analysis to determine the association of metabolic syndrome status with each group after adjusting for age, smoking, and exercise frequency, the odds ratios (ORs) in the NAFLD group (OR = 2.397, *P* = 0.002) and AFLD group (OR = 4.445, *P* = 0.001) were significantly higher than that in the healthy group (Table 3), but no significant difference was found between the NAFLD and AFLD groups (Table 4).

**Table 3:** Relationship between the study subjects^a^ and metabolic syndrome

Metabolic Syndrome	OR	*P* value^b^	95% Confidence interval
Variable			
Healthy	1		
NAFLD	2.397	0.002	1.365–4.208
AFLD	4.445	0.001	1.792–11.022

*Abbreviations*: *NAFLD* non-alcoholic fatty liver disease, *AFLD* alcoholic fatty liver disease, *OR* odds ratio

^a^Adjusted for age, smoking quantity, and exercise frequency

^b^The *P* values were calculated using the multiple logistic regression test

**Table 4:** Relationship between the type of fatty liver^a^ and metabolic syndrome

Metabolic Syndrome	OR	*P* value^b^	95% Confidence interval
Type of fatty liver			
AFLD	1		
NAFLD	0.565	0.226	0.225–1.423

*Abbreviations*: *NAFLD* non-alcoholic fatty liver disease, *AFLD* alcoholic fatty liver disease, *OR* odds ratio

^a^Adjusted for age, smoking quantity, and exercise frequency

^b^The *P* values were calculated using the multiple logistic regression test

## Discussion

Fatty liver disease has the highest prevalence rate among liver diseases not only worldwide but also in South Korea. According to a recent study conducted in South Korea, the prevalence rate of fatty liver disease diagnosed using ultrasonography was 30%, accounting for 69.7% of liver disease cases [16]. Similarly, the morbidity rate of fatty liver disease in this study was 32.3%. The worldwide prevalence rate of NAFLD varies between studies [14]. In the United States, one-third have been diagnosed with NAFLD, and in the United Kingdom it is reported as 29 cases per 100,000 person-years, which is clearly on a globally increasing trend [17, 18].

Because it is a standard commonly used in the diagnosis of NAFLD, the weekly alcohol consumption among patients diagnosed with NAFLD ranges from 0 to 140 g for men or 0 to 70 g for women [14, 15]. In this study, we defined nondrinkers as individuals who consumed < 140 g per week, according to the standard proposed by Choi et al. [13]. In addition, while the total drinking quantity was measured through the questionnaire, 350 mL of beer, 120 mL of wine, 25 mL of brandy, or 50 mL of soju was considered to contain 10 g of alcohol by surveying the type of alcohol and the number of bottles consumed.

There was no difference between the NAFLD and AFLD groups for each of the indicators of cardiovascular risk factors. Importantly, however, these indicators differed between those two groups and the healthy group. This conclusion suggests that the risk factors are more related to the presence or absence of fatty liver, regardless of the cause of the occurrence of fatty liver. Systolic blood pressure, diastolic blood pressure, fasting plasma glucose level, total cholesterol level, and TG level were higher, whereas high-density cholesterol was lower in the NAFLD group than in the healthy group, indicating an increase in cardiovascular risk indicators in the NAFLD group. NAFLD is reportedly accompanied by obesity (30–100%), diabetes (10–75%), or dyslipidemia (20–92%) [14]. In a study on the association between NAFLD and cardiovascular disease, blood pressure, fasting plasma glucose level, total cholesterol level, and TG level were higher in the NAFLD group than in the healthy group. In another study [19], systolic blood pressure, fasting plasma glucose level, total cholesterol level, and TG level were higher in the NAFLD group than in the healthy group, although sex-related differences in the values were found. The previous results are similar to the results of this study.

Diastolic blood pressure, fasting plasma glucose level, total cholesterol level, TG level, and hsCRP level were all higher in the AFLD group than in the healthy group. That is, except for high-density cholesterol levels, the values of the cardiovascular indicators in the AFLD group, as in the NAFLD group, were higher than those in the healthy group. Studies on the relationship between high-density cholesterol levels and fatty liver disease reported that high-density cholesterol levels were decreased in fatty liver [20, 21], though one study reported a contradictory result [22]. Additionally, other studies reported that low-density cholesterol levels were decreased in NAFLD [23, 24]. In this study, low-density cholesterol levels were higher in the NAFLD group than in the healthy group. Several studies that investigated TG levels reported, as found in this study, that TG increased in both the NAFLD and AFLD groups [20–22, 25]. Although no difference was found in the hsCRP level, one of the cardiovascular risk indicators, in both the healthy and NAFLD groups, was lower than that in the AFLD group. Among the many studies that support the association between fatty liver disease and inflammation indicators, one identified that high hsCRP levels were independently associated with fatty liver disease, obesity, and metabolic syndrome [25], whereas another reported that hsCRP level was a strong predictor of NAFLD [26].

While the metabolic syndrome rate was higher in the NAFLD and AFLD groups than in the healthy group, no significant difference was observed between the NAFLD and AFLD groups. A previous study reported that the fatty acid content in the liver was increased in patients with metabolic syndrome regardless of age, sex, or BMI [27], and an association between several metabolic disorders and serious liver diseases was identified [28, 29].

As for the limitations of this study, the study results cannot be generalized because all the patients included in the study were hospitalized at the same health promotion center. Moreover, achieving reliability was difficult because the alcohol consumption level, smoking habit, and exercise frequency were surveyed through a self-administered questionnaire. In addition, NAFLD was determined by CT with no histologic confirmation of fatty liver. However, the fact that the AST/ALT ratio in most NAFLD patients was below 1 rationalizes the selection method of patients for the NAFLD group in this study [30]. Another limitation is as follows. NAFLD was renamed metabolic dysfunction associated fatty liver disease (MAFLD) [31–33]. Because NAFLD is an “exclusive” disease, it is insufficient to explain its pathogenesis and management. The diagnostic criteria for MAFLD better reflect disease mechanisms than those for NAFLD [31–33]. However, we designed this study before MAFLD was termed. In the future, there is a need to develop this research on the subject using MAFLD.

This is a comparative study on not only NAFLD, which has been previously studied extensively, but also on AFLD, and patients taking medications for hypertension, diabetes, hyperlipidemia, ischemic heart disease, myocardial infarction, cerebral infarction, and hemorrhage were excluded. Therefore, this study is significant in that it provided a pure association between the cardiovascular risk indicators and the NAFLD or AFLD group.

## Conclusions

The values of the cardiovascular risk indicators were significantly higher in the NAFLD and AFLD groups than in the healthy group in this study. Ultimately, considering that the patients in both the NAFLD and AFLD groups have the same cardiovascular disease risks, patients diagnosed with fatty liver disease should be examined for risk factors of cardiovascular diseases and should undergo management and treatment of such diseases. Although this study demonstrated that cardiovascular risk indicators and the severity of fatty liver are not associated, additional studies in this area are necessary.

## Supplementary Information

**Additional file 1.**

## Data Availability

Not applicable.

## Abbreviations

AFLD: Alcoholic fatty liver disease
NAFLD: Non-alcoholic fatty liver disease
CT: Computed tomography
MRI: Magnetic resonance imaging
hsCRP: high-sensitivity C-reactive protein
AST: Aspartate aminotransferase
ALT: Alanine aminotransferase
ϓ-GT: Gamma-glutamyl transpeptidase
BMI: Body mass index
HU: Hounsfield units
OR: Odds ratio
TG: Triglycerides
